# Cellular hnRNP AB inhibits avian influenza virus RNA synthesis via blocking UAP56-mediated nuclear export of PB2 mRNA

**DOI:** 10.1186/s13567-025-01660-3

**Published:** 2025-11-21

**Authors:** Shuhui Liu, Yue Sun, Yunling Peng, Chenchen Xu, Suquan Song, Liping Yan

**Affiliations:** MOE Joint International Research Laboratory of Animal Health and Food Safety, Jiangsu Engineering Laboratory of Animal Immunology, Jiangsu Detection Center of Terrestrial Wildlife Disease, Institute of Immunology, College of Veterinary Medicine, Nanjing Agricultural University, Nanjing, 210095 Jiangsu Province China

**Keywords:** Avian influenza virus, hnRNP AB, PB2, vRNA synthesis, nuclear export

## Abstract

Avian influenza viral ribonucleoproteins (vRNPs) complete genome transcription and replication by interacting with host proteins, and RNA-dependent RNA polymerase (RdRp) is its major component. PB2 is a component of RdRp and plays an important role in viral RNA synthesis. Our previous mass spectrometry analysis identified PB2 interacted with avian cellular heterogeneous nuclear ribonucleoprotein AB (hnRNP AB). However, the specific mechanism of this interaction regulating viral replication needs to be further clarified. In this study, we found that avian hnRNP AB inhibited the replication of multiple subtypes of avian influenza viruses (AIVs) from different reservoirs, and the glycine-rich domain (GRD) of hnRNP AB was the function domain that inhibited AIV replication. Moreover, we demonstrated that the GRD of avian hnRNP AB interacted with the C-terminus of PB2, reducing the binding of PB1 to PB2 and interfering with RdRp assembly. Based on the previous discovery that hnRNP AB affected the nucleoplasmic distribution of PB2 mRNA, we have further explored the mechanism here. Mechanically, hnRNP AB intervened in the nuclear export of PB2 mRNA by reducing the binding ability of UAP56, and decreased PB2 expression to interfere with RdRp formation and reduce vRNA synthesis, which in turn inhibited viral replication. Collectively, this study demonstrated that the avian host protein hnRNP AB inhibited AIV replication by blocking assembly of RdRp and vRNA synthesis, in which was associated with UAP56-mediated nuclear export of PB2 mRNA, providing a potential target for antiviral intervention.

**Graphical Abstract:**

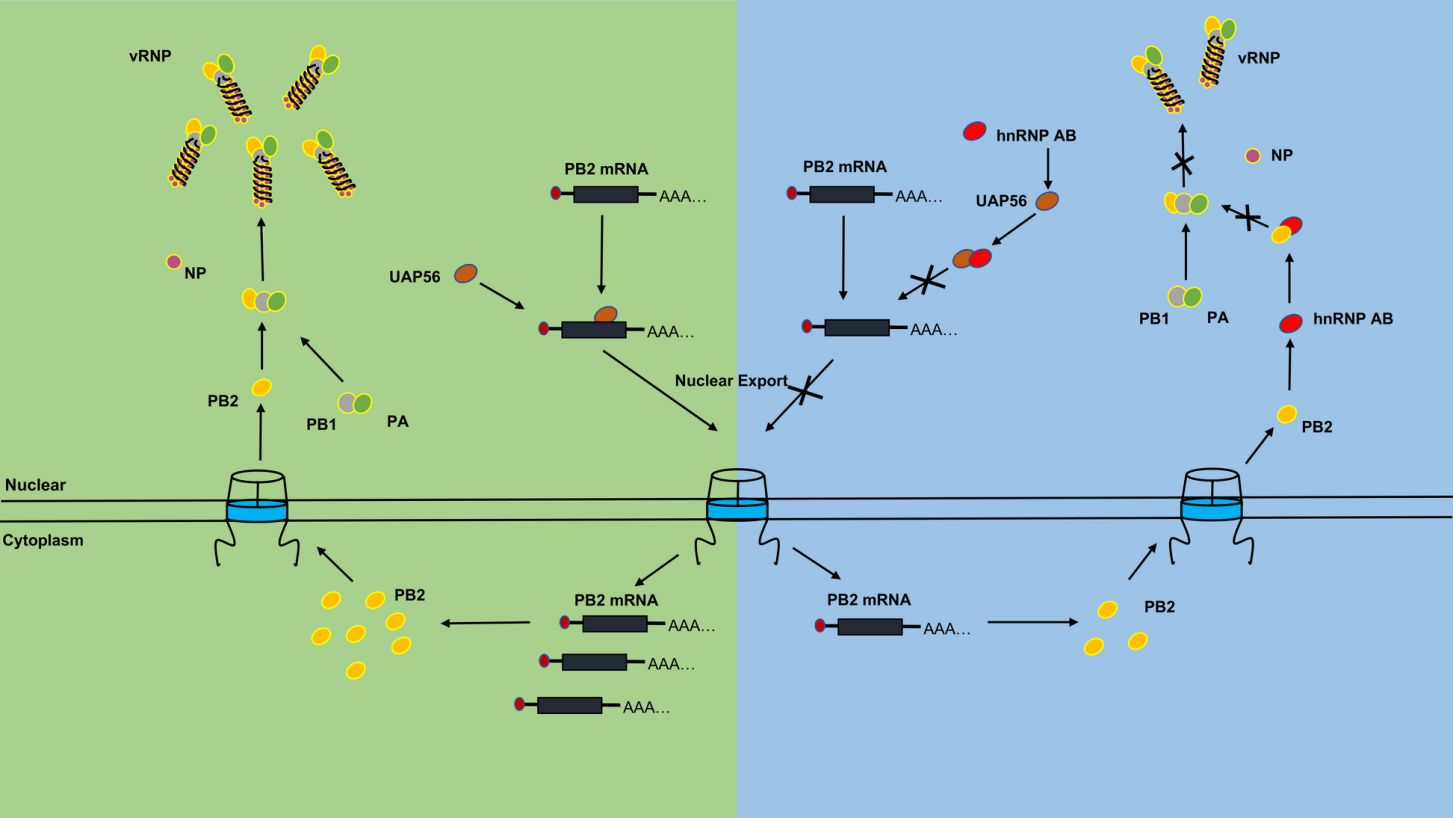

## Introduction

Avian influenza virus (AIV) is an important pathogen with the potential for rapid evolutionary change and cross-species transmission, posing a serious threat to public health [1]. The viral ribonucleoprotein (vRNP), the core of AIV replication and transcription, consists of the polymerase complex, viral genome and nucleoproteins (NPs) [2]. The vRNP is transferred to the nucleus of AIV-infected host cell, where influenza virus polymerase carries out viral RNA synthesis. The PB2 is the main component of the RNA-dependent RNA polymerase (RdRp). RdRp uses its distinctive cap binding complex to capture the 5’ end of nascent host RNA as a primer for transcription, and completes mRNA transcription through the host cellular machinery [3, 4]. In addition, the E627K substitution in the PB2 protein affects the host range and pathogenicity [5, 6]. This implies that PB2 may be an important target for host defense against AIV attack.

AIVs rely on interactions with host factors to regulate their own life cycle. Heterogeneous nuclear ribonucleoproteins (hnRNPs) are often considered to be one of the most important RNA binding proteins (RBP) family involved in the viral life cycle [7]. Some members of hnRNPs inhibit influenza A virus (IAV) replication by interacting with viral proteins or RNAs, blocking nucleocytoplasmic transport of viral mRNA or splicing of pre-mRNA [8–11]. Of note, chicken hnRNP M specifically inhibits M2 mRNA splicing to maintain proper M2 protein levels, which in turn promotes IAV replication in avian cells [12]. These species-specific regulatory mechanisms highlight the complex roles of hnRNPs in virus replication. Particularly, hnRNP AB belongs to the hnRNP A/B family [13], which is a negative regulator of influenza virus replication. For example, it has been reported that hnRNP AB can interact with NPs to limit the nuclear export of viral mRNA by preventing the binding of mRNA to nuclear export factors [14]. HnRNP AB also targets NPs to disrupt the conformation and activity of the RdRp complex [15]. Our previous studies have found that hnRNP AB interacted with PB2 protein and restricted viral replication [16], but the specific mechanism needs to be further explored.

Our previous research has shown that hnRNP AB inhibited PB2 protein expression by reducing PB2 mRNA nuclear export [16]. The transcription-export (TREX) plays an important role in mRNA nuclear export, including the THO complex, UAP56, AML-1 and LEF-1 (ALY) and other important proteins [17, 18]. UAP56 acts as a splicing factor that interacts with pre-mRNA, and subsequently recruits ALY to the mRNA. Then, mRNA is transferred to the nuclear pore complex (NPC) by downstream nuclear export factors, completing the process of mRNA nuclear export [19]. The IAV mRNA nuclear export is associated with UAP56, while the mRNA nuclear export pathway of AIV in avian hosts has not been fully investigated [20, 21]. In addition, hnRNPs are nucleoplasmic shuttle proteins and involved in mRNA nuclear export [22]. Here, we speculated that hnRNP AB might hijack nuclear export factor to inhibit PB2 mRNA nuclear export and restrict AIV replication.

In this study, we demonstrated that the avian host protein hnRNP AB could inhibit the replication of multiple subtypes AIVs from different host sources, with the glycine-rich domain (GRD) as the functional domain. The GRD of hnRNP AB interacted with the C-terminus of PB2 and interfered with the interaction between PB2 and PB1. Meanwhile, hnRNP AB inhibited PB2 mRNA nuclear export by interacting with the host nuclear export factor UAP56, inhibited PB2 protein expression, and ultimately reduced viral RNA synthesis. In summary, hnRNP AB targeted PB2 to inhibit the formation of RdRp, thereby reducing the RNA synthesis in AIV replication.

## Materials and methods

### Cells, viruses and antibodies

DF-1 cells, HEK293T cells, and MDCK cells were cultured using Dulbecco’s modified Eagle’s medium (C11965500BT, Gibco) with 10% fetal bovine serum (c04001, Vivacell). The culture medium was treated with a final concentration of 100 U of penicillin and 0.1 mg/mL of streptomycin (c0222, Beyotime). The cells were maintained in an incubator at 37 ℃ with containing 5% CO_2_, A/chicken/Zhejiang/A2013/2017 (H9N2), A/Anser fabalis/Jiangsu/G1833/2023 (H9N2), A/Anser fabalis/Jiangsu/G721/2023 (H4N6) and A/Black-headed Gull/Jiangsu/F463/2022 (H13N3) were available in our laboratory. The viruses were proliferated using 9-day-old specific pathogen free chicken embryos. Antibodies used in this study are as follows: anti-Flag mouse monoclonal antibody (F1804, Sigma), anti-Myc rabbit polyclonal antibody (R1208-1, Hua An Biotechnology), anti-hnRNPAB rabbit polyclonal antibody (D164202-0025, Sanon Biotech), anti-PB2 rabbit polyclonal antibody (GTX125926-S, GeneTex), anti-NP mouse monoclonal antibody (IT-003-023M1, Cambridge biologics), anti-GAPDH mouse monoclonal antibody (60004-1-Ig, Proteintech), anti-Vinculin rabbit polyclonal antibody (BA2934, BOSTER), anti-Lamin B rabbit polyclonal antibody (BA1228, BOSTER), FITC-anti-mouse goat polyclonal antibody (BL031A, Biosharp), HRP-anti-mouse goat polyclonal antibody (074-1806, KPL) and HRP-anti-rabbit goat polyclonal antibody (074-1506, KPL) were purchased from commercial sources.

### Plasmids

The pCAGGS-Flag, pCAGGS-Myc, Flag-hnRNP AB and Myc-PB2 plasmids were stored in our laboratory. Linearized the Flag-pCAGGS and Myc-pCAGGS vector using *Xhol* I (1635, Takara) and *EcoR* I (1611, Takara) enzymes. Truncates were constructed based on the PB2 proteins of A/chicken/Zhejiang/A2013/2017 (H9N2) and hnRNP AB (NM_205328.6), respectively. The ORF regions of PB1 (A/chicken/Zhejiang/A2013/2017), hnRNP AB (NM_205328.6) and UAP56 (XM_040695300.1) were homologously recombined into the pCAGGS-Myc expression vector (C116, Vazyme). The ORF region of PB2 (A/chicken/Zhejiang/A2013/2017) was homologously recombined into pCAGGS-Flag expression vector (C116, vazyme). The specific primers are shown in the Table 1.

**Table 1:** **Primers used in this study**

Primer	Sequence (5′–3′)
*Construction of expression vectors*	
Flag-ΔCBFNT-F	*acaaggacgacgatgacaag*ATGGAGGAGGACGCGGGGAAAATG
Flag-ΔCBFNT-R	*gagggaaaaagatctgctag*TCAATATGGCTTGTAGTTATTC
Flag-RRMs-F	*acaaggacgacgatgacaag*ATGGAGGAGGACGCGGGGAAAATG
Flag-RRMs-R	*gagggaaaaagatctgctag*TCATGCTACCTTTATCTCGCAC
Flag-ΔGRD-F	*acaaggacgacgatgacaag*ATGTCCGAAGCGGAGCAGCAG
Flag-ΔGRD-R Flag-GRD-F Flag-GRD-R	*gagggaaaaagatctgctag*TCATGCTACCTTTATCTCGCAC *acaaggacgacgatgacaag*ATGCAGCCAAAAGAAGTATACCAGC *gagggaaaaagatctgctag*TCAATATGGCTTGTAGTTATTCTGA
Flag-PB2-F	*acaaggacgacgatgacaag*ATGGAAAGAATAAAAGAACTAAGAG
Flag-PB2-R	*gagggaaaaagatctgctag*TTAATTGATGACCATCCGAATC
Myc-PB2-N-F	*tcatctctgaagaggatctg*ATGGAAAGAATAAAAGAACTAAG
Myc-PB2-N-R	*gagggaaaaagatctgctag*TTACTGTTCTTCTGTTGGGTTTTGTC
Myc-PB2-M-F	*tcatctctgaagaggatctg*ATGGCTGTGGATATATGTAAGG
Myc-PB2-M-R	*gagggaaaaagatctgctag*TTAGGACGATGAATATGTTATAG
Myc-PB2-C-F	*tcatctctgaagaggatctg*ATGATGTGGGAGATCAATGGT
Myc-PB2-C-R	*gagggaaaaagatctgctag*TTAATTGATGACCATCCGAATC
Myc-PB1-F	*tcatctctgaagaggatctg*ATGGATGTCAATCCGACTTTACT
Myc-PB1-R	*gagggaaaaagatctgctag*CTATTTTTGCCGTCTGAGCTCTT
Myc-hnRNP AB-F	*tcatctctgaagaggatctg*ATGTCCGAAGCGGAGCAGCAGT
Myc-hnRNP AB-R	*gagggaaaaagatctgctag*TCAATATGGCTTGTAGTTATTC
Myc-UAP56-F	*tcatctctgaagaggatctg*ATGGCGGAGAACGACGTGGACAACG
Myc-UAP56-R	*gagggaaaaagatctgctag*TTATCGGGTCTGCTCGATGTAGGA
*siRNA*	
sihnRNP AB-sense	GGCGACCAGAUCAACGCCAGC
sihnRNP AB-antisense	UGGCGUUGAUCUGGUCGCCUU
NC-sense	UUCUCCGAACGUGUCACGUTT
NC-antisense	ACGUGACACGUUCGGAGAATT
*RIP for PB2 mRNA*	
RIP-F	ATGGAAAGAATAAAAGAACTAAG
RIP-R	TCATCCCGGTGTGTACATTTG
*Reverse transcription*	
12uni for vRNA	AGCAAAAGCAGG
13uni for cRNA	AGTAGAAACAAGG
Oligo d(T)_18_ for mRNA	TTTTTTTTTTTTTTTTTT
*qRT-PCR for mRNAs*	
NP-F	ATTTTTCTGGCAAGGTCTGCACTC
NP-R PB2-F PB2-R	TAAAGACCTGGCTGTTTTGAAGCAG AGAGCAACGGTATCAGC CGTCTTTCCCACTCACTATC
GAPDH-F	AAGCTGTGGAGAGATGGCAGA
GAPDH-R	TATCATCATACTTGGCTGGTT
UAP56-F	GGGCAGGTATCGGTGTTGG
UAP56-R	CTTCAGGTTGAGGCTCTTATTGC

The homologous arms of the plasmids are indicated in italics in the primer sequence

### Transfection

DF-1 cells or 293 T cells were transfected at 80% confluence. JetPrime (101000046, polyplus) was used according to the instructions, the plasmid and transfection reagent were evenly added to each well of cells. The 6-well plate was placed in the cell culture incubator to continue culture. After 6–8 h of transfection, the maintenance medium was replaced.

### siRNA transfection

DF-1 cells were transfected at 50% confluence. JetPrime (101000046, polyplus) was used according to the instructions, the siRNA targeting hnRNP AB and transfection reagent were evenly added to each well of cells. The 12-well plate was placed in the cell culture incubator to continue culture. After 6–8 h of transfection, the maintenance medium was replaced. The sequences of siRNAs targeting hnRNPAB (Generay, China) were listed in Table 1.

### Co-immunoprecipitation

The cell was lysed with cell lysate (P0013, Beyotime) containing Cocktail (HY-K0010, MCE), and the supernatant was centrifuged and added to proteinA/G agarose beads (sc-2003, santa cruz), and mixed at 4 ℃ for 30 min. After centrifugation, the supernatant was added with 3 μg anti-Flag tag or anti-Myc tag antibody. The mixture was mixed at 4 °C for more than 4–6 h, then agarose beads were added and mixed. After sufficient binding, the mixture was centrifuged and the supernatant was discarded and the precipitate was washed with lysate.

### Western blot

The extracted cellular proteins were made into samples, and the samples were separated by SDS-PAGE and transferred to a PVDF membrane (PVH00010, Merck Millipore). The PVDF membrane was blocked in 5% skimmed milk. After blocking, the PVDF membrane was incubated with primary antibody overnight at 4 °C. Then the PVDF membrane was incubated with HRP-labelled secondary antibody at 37 °C for 1 h. Bands were detected using enhanced chemiluminescence (E432, Vazyme).

### Indirect immunofluorescence assays

MDCK cells were seeded in 96-well cell culture plates for virus infection. The virus solution was diluted tenfold. After 2 h of induction in a 37 °C cell culture incubator, the viral solution was discarded and 2% maintenance solution was added and incubated for 72 h. 4% paraformaldehyde was added to fix the cells, and the cell plates were washed with PBS and blocked with 5% skimmed milk. After blocking, anti-NP protein antibody was incubated at 4 °C overnight. Fluorescent secondary antibody was incubated at 37 °C for 1.5 h in the dark. After the secondary antibody incubation, fluorescence was observed. All results should be obtained based on the establishment of a negative control. The Reed-Muench method was used to calculate TCID_50_.

### RNA extraction, reverse transcription and qRT-PCR

Total RNA of treated DF-1 cells was extracted using Trizol (R401-01, Vazyme), referring to the instruction manual. The extracted equivalent concentration (1 μg) of RNA was used for reverse transcription to obtain cDNA. The first strand cDNA was synthesised using the Rapid Reverse Transcription Kit (11149ES10, Yeasen), and the corresponding cDNA was obtained by reverse transcription using specific primers respectively. The cDNA was subjected to real-time PCR by Hieff UNICON qPCR SYBR Green Master Mix (11198ES08, Yeasen) using specific primers, and the procedure was set up as described in the instruction manual (Quant Gene 9600). A single melting peak was observed for the products of each primer pair. Amplification efficiencies of each primer ranged from 90 to 110%. The relative RNA levels were calculated by the 2^−ΔΔCT^ method, including normalization to CT values of GAPDH. The specific primers are shown in the Table 1.

### Nuclear and cytoplasmic fractionation

Cytoplasmic and nuclear fractions were separated using Nuclear and Cytoplasmic Protein Extraction Kit (P0028, Beyotime) according to the instruction.

### RNA immunoprecipitation

The precipitate of treated DF-1 cell was collected. Immunoprecipitation was performed with anti-Myc tag antibody, RNA in IP samples was extracted by Trizol method. RNA was reverse transcribed into cDNA and PB2 mRNA was detected by RT-PCR and qRT-PCR.

### Statistical analysis

All experiments were repeated at least three times. The data images were processed using Image J and then GraphPad Prism analyzed the research data and generated statistical analysis graphs, unpaired two-tailed t-test was used to analyze differences between data. Significant differences were considered at * *p* < 0.05, ** *p* < 0.01 or *** *p* < 0.001 respectively.

## Results

### Overexpression of hnRNP AB inhibits replication of different subtypes of AIVs

**Figure 1:**
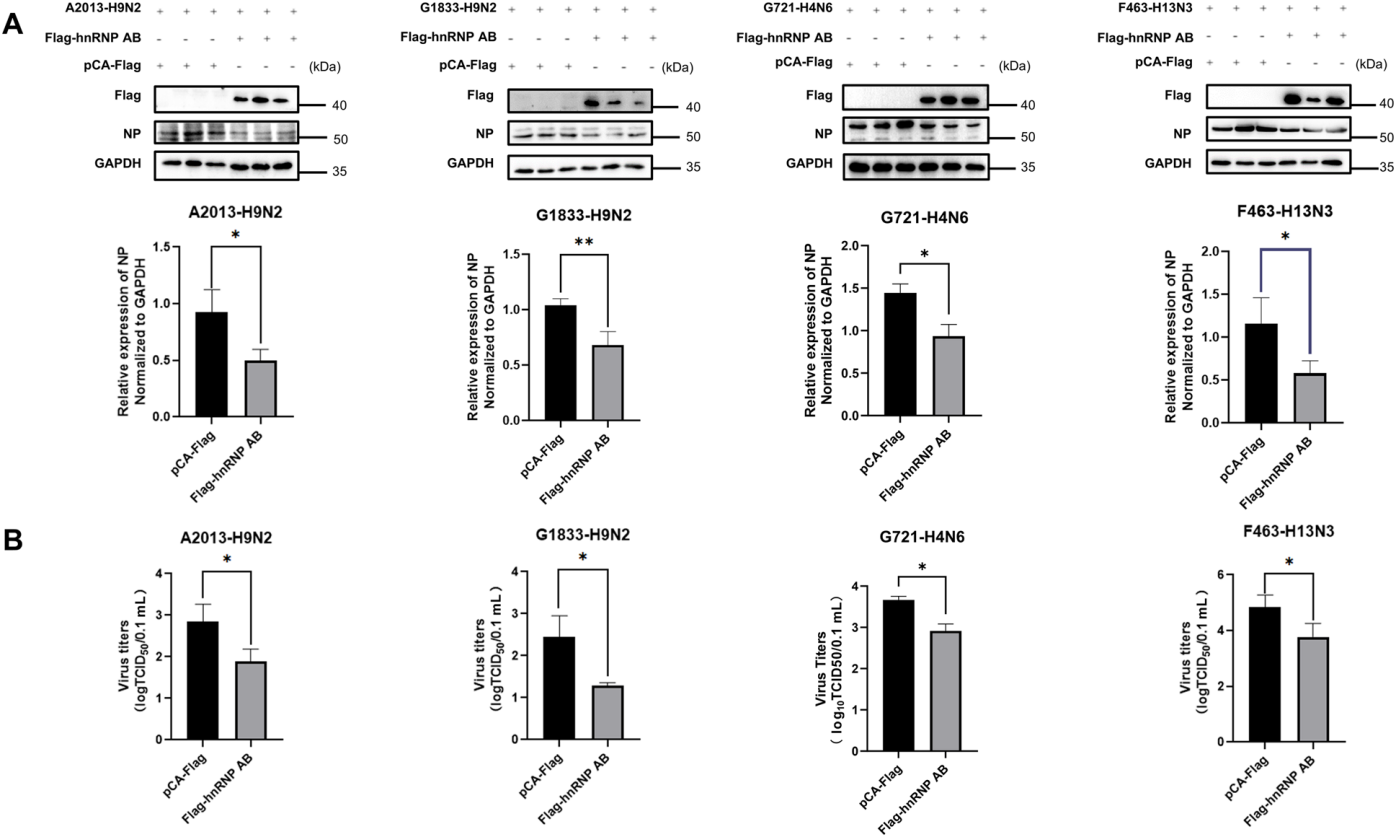
**Overexpression of hnRNP AB inhibits replication of different subtypes of AIVs**. **A** Overexpression of hnRNP AB can reduce the NP proteins of AIVs from different sources and different subtypes. DF-1 cells were transfected with pCA-Flag or Flag-hnRNP AB for 24 h, then they were infected with A2013-H9N2, G1833-H9N2, G721-H4N6 and F463-H13N3 for 24 h (MOI of 0.01), respectively. Cell lysates were collected at 24 hpi. Western blot was used to detect the expressions of Flag-hnRNP AB, NP and GAPDH. **B** Overexpression of hnRNP AB can reduce AIV titers from different sources and subtypes. DF-1 cells were transfected with pCA-Flag or Flag-hnRNP AB for 24 h, then they were infected with A2013-H9N2, G1833-H9N2, G721-H4N6 and F463-H13N3 for 24 h (MOI of 0.01), respectively. Cell supernatants were collected at 24 hpi. IFA was used to determine viral titer. The experiment was repeated three times, the expression level of viral NP protein was normalized to GAPDH using ImageJ respectively, all by two-tailed Student’s *t* test. * *p* < 0.05, ** *p* < 0.01.

According to previous studies, hnRNP AB has an inhibitory effect on H9N2 (A2013) [16]. We further explored whether the negative regulatory effect of hnRNP AB on AIVs is universal. The hnRNP AB was overexpressed in DF-1 cells and cells were infected with H9N2 (A2013), H9N2 (G1833), H4N6 (G721) and H13N3 (F463) viruses, respectively. A2013 was isolated from poultry. G1833, G721, and F463 were isolated from wild birds. As shown in Figure 1A, overexpression of hnRNP AB reduced the expression of viral NP protein of A2013, G1833, G721 and F463, compared with the control group. Figure 1B showed that overexpressing hnRNP AB group had a lower viral titer than the control group. These results suggested that the inhibitory effect of hnRNP AB on AIV replication was universal.

### Endogenous hnRNP AB inhibits replication of different subtypes of AIVs

**Figure 2:**
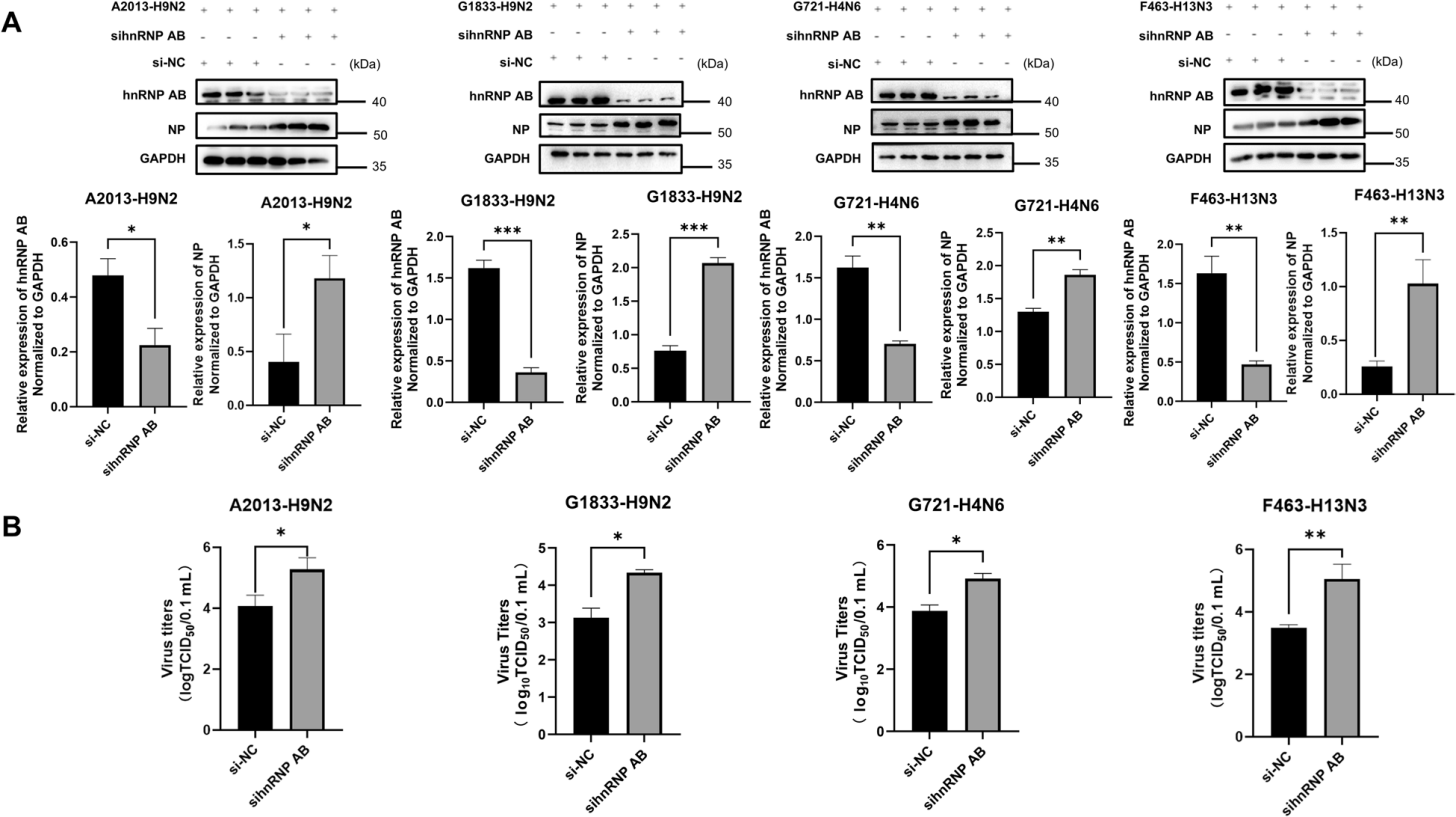
**Endogenous hnRNP AB inhibits replication of different subtypes of AIVs.** **A** Endogenous hnRNP AB can reduce the NP proteins of AIVs from different sources and different subtypes. DF-1 cells were transfected with si-NC or sihnRNP AB for 24 h, then they were infected with A2013-H9N2, G1833-H9N2, G721-H4N6 and F463-H13N3 for 24 h (MOI of 0.01), respectively. Cell lysates were collected at 24 hpi. Western blot was used to detect the expressions of hnRNP AB, NP and GAPDH. **B** Endogenous hnRNP AB can reduce AIV titers from different sources and subtypes. DF-1 cells were transfected with si-NC or sihnRNP AB for 24 h, then they were infected with A2013-H9N2, G1833-H9N2, G721-H4N6 and F463-H13N3 for 24 h (MOI of 0.01), respectively. Cell supernatants were collected at 24 hpi. IFA was used to determine viral titer. The experiment was repeated three times, the expression levels of hnRNP AB and viral NP protein were normalized to GAPDH using ImageJ respectively, all by two-tailed Student’s *t* test. * *p* < 0.05, ** *p* < 0.01, *** *p* < 0.001.

We further verified the effect of the host protein hnRNP AB on AIVs replication by interfering with the expression of the endogenous hnRNP AB protein. DF-1 cells were transfected with sihnRNP AB and cells were infected with H9N2 (A2013), H9N2 (G1833), H4N6 (G721) and H13N3 (F463) viruses, respectively. As shown in Figure 2A, the sihnRNP AB group elevated the expression of A2013, G1833, G721, and F463 viral NP proteins compared to the control group. Figure 2B showed that the viral titer of the sihnRNP AB group was higher than that of the control group. These results suggested that endogenous hnRNP AB inhibited replication of different subtypes of AIVs from different host sources.

### The GRD is the functional domain of hnRNP AB that inhibits AIV replication

**Figure 3:**
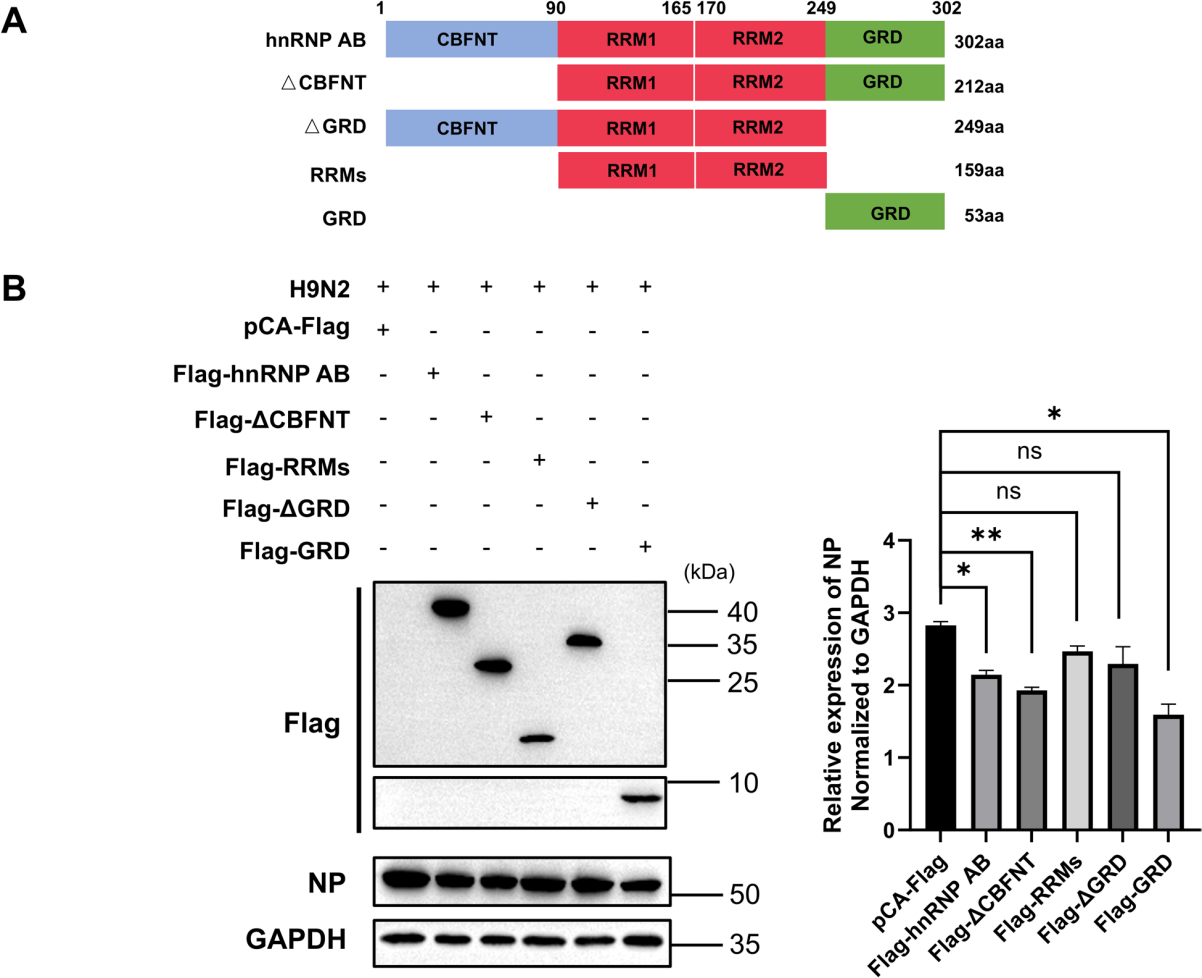
**The GRD region is the functional domain of hnRNP AB to inhibit AIV**. **A** Schematic representation of the truncated forms of hnRNP AB. **B** DF-1 cells were transfected with pCA-Flag, Flag-hnRNP AB, Flag-ΔCBFNT, Flag-RRMs, Flag-ΔGRD and Flag-GRD for 24 h, respectively. Then the transfected cells were infected with A2013-H9N2 (MOI of 0.01) for 24 h. Cell lysates were collected at 24 hpi. Western blot was used to detect the expressions of Flag-hnRNP AB, Flag-ΔCBFNT, Flag-RRMs, Flag-ΔGRD, Flag-GRD, NP and GAPDH. The experiment was repeated three times, the expression level of viral NP protein was normalized to GAPDH using ImageJ respectively, all by two-tailed Student’s *t* test. * *p* < 0.05, ** *p* < 0.01.

The structure of hnRNP AB contains four domains: the N-terminal CARG-binding factor-A (CBFNT), the two RNA recognition motif (RRMs) in the middle, and the GRD at the C-terminus. ΔCBFNT, RRMs and ΔGRD truncates of hnRNP AB were constructed to explore functional domain. As shown in Figure 3, full-length hnRNP AB, ΔCBFNT and GRD domain significantly reduced the expression of NP, while RRMs or ΔGRD did not reduce the expression of NP. These results suggested that the GRD region was the functional domain of hnRNP AB to inhibit AIV replication.

### The GRD of hnRNP AB interacts with the C-terminus of PB2, and interfered with PB1-PB2 interactions

**Figure 4:**
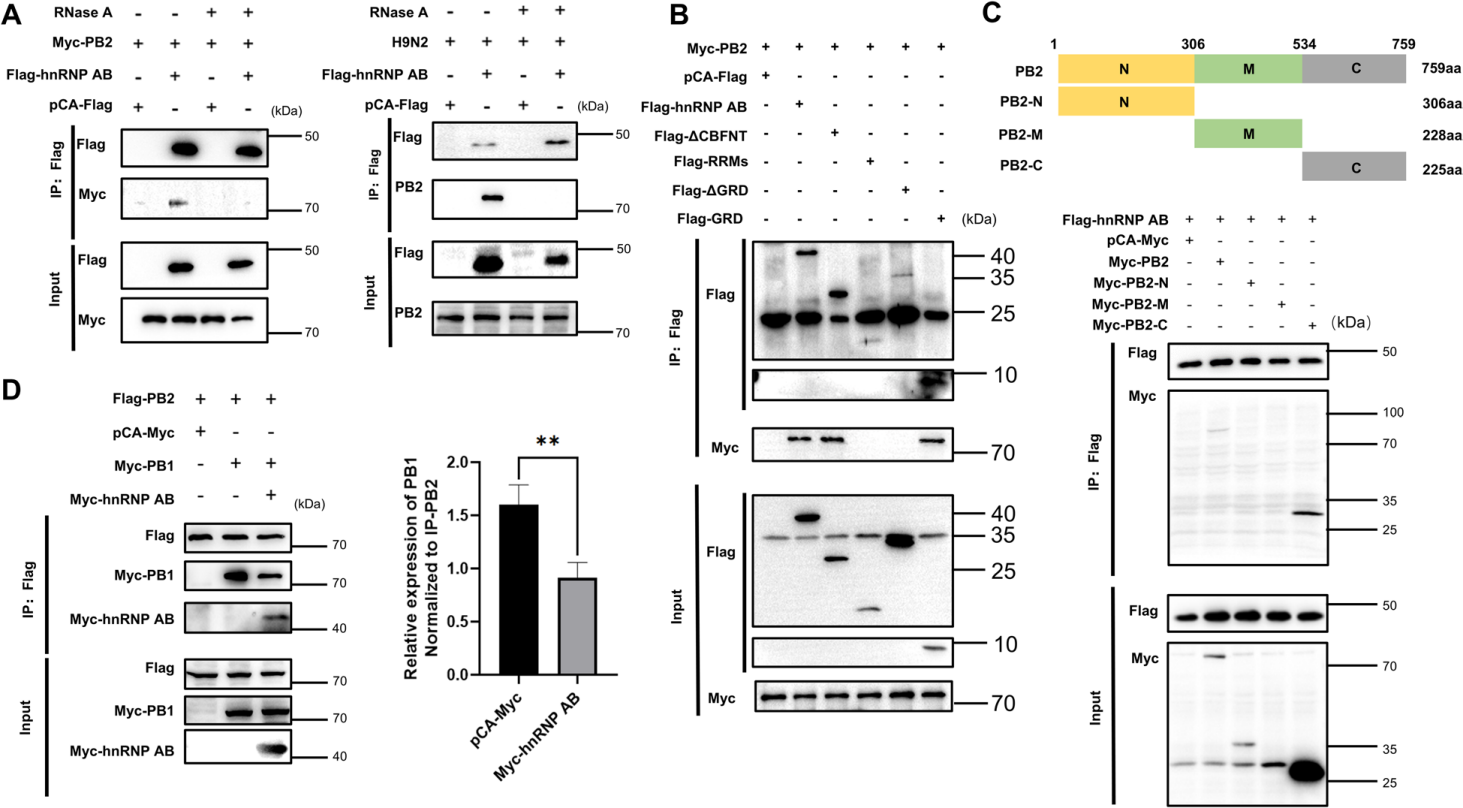
**The GRD of hnRNP AB interacts with the C-terminus of PB2**. **A** RNA mediates interaction of PB2 and hnRNP AB. 293 T cells were co-transfected with Myc-PB2 and Flag-hnRNP AB (pCA-Flag) for 48 h, and cell lysates were collected. DF-1 cells were transfected with Flag-hnRNP AB (pCA-Flag) for 24 h. Then the transfected cells were infected with A2013-H9N2 (MOI of 0.1) for 24 h. Cell lysates were collected at 24 hpi, RNase treated, and co-immunoprecipitated with anti-Flag tag antibody. Western blot was used to analyze the protein contents of Flag-hnRNP AB, Myc-PB2 and PB2. **B** GRD is the interaction domain. 293 T cells were co-transfected with different truncates of Flag-hnRNP AB (pCA-Flag) and Myc-PB2 for 48 h, respectively. Cell lysates were collected, and co-immunoprecipitated with anti-Flag tag antibody. Western blot was used to detect Flag-hnRNP AB truncates and Myc-PB2. **C** PB2-C is the interaction domain. 293 T cells were co-transfected with different truncates of Myc-PB2 (pCA-Myc) and Flag-hnRNP AB for 48 h, respectively. Cell lysates were collected, and co-immunoprecipitated with anti-Flag tag antibody. Western blot was used to detect Flag-hnRNP AB and Myc-PB2 truncates. **D** Avian host protein hnRNP AB inhibits PB2-PB1 interaction. 293 T cells were co-transfected with Flag-PB2 and pCA-Myc, Flag-PB2 and Myc-PB1, Flag-PB2, Myc-PB1 and Myc-hnRNP AB for 48 h, respectively. Cell lysates were collected and co-immunoprecipitated with Flag-tag antibody. Western blot was used to detect Flag-PB2, Myc-PB1 and Myc-hnRNP AB proteins. The level of Myc-PB1 protein were normalized to Flag-PB2 using ImageJ respectively. The experiment was repeated three times, all by two-tailed Student’s *t* test. ** *p* < 0.01.

HnRNP AB is an RNA-binding protein, and PB2 is a major component of RdRp, which is involved in the modification and processing of viral RNA [23]. Here, the interaction between hnRNP AB and PB2 was further explored. Firstly, it was tested whether the interaction between hnRNP AB and PB2 was dependent on RNA. The results of Figure 4A showed that hnRNP AB immunoprecipitated to PB2 in both endogenous and exogenous experiments, but the interaction disappeared after RNase treatment, indicating that the interaction between hnRNP AB and PB2 was dependent on RNA.

The key interaction domains between hnRNP AB and PB2 were further explored. As shown in Figure 4B, Flag-hnRNP AB, Flag-ΔCBFNT and Flag-GRD were successfully immunoprecipitated to Myc-PB2, and the CBFNT domain was not necessary for the interaction between hnRNP AB and PB2. Flag-RRMs and Flag-ΔGRD were not immunoprecipitated to PB2, indicating that GRD domain was the interaction domain between hnRNP AB and PB2. The PB2 protein consists of 759 amino acids, which we divided into three structural domains (PB2-N, PB2-M, and PB2-C truncation) for study. In this experiment, only Myc-PB2 and Myc-PB2-C were co-immunoprecipitated with hnRNP AB, indicating the C-terminus of PB2 was the region that interacted with hnRNP AB (Figure 4C).

The C-terminal of PB2 is also a potential binding domain with PB1 [24]. Therefore, we explored if hnRNP AB affected PB2-PB1 interaction. As shown in Figure 4D, co-transfection of Myc-hnRNP AB reduced the amount of Myc-PB1 immunoprecipitated to Flag-PB2. It indicated that hnRNP AB interfered with the PB2-PB1 interaction.

### HnRNP AB inhibits AIV RNA synthesis levels

**Figure 5:**
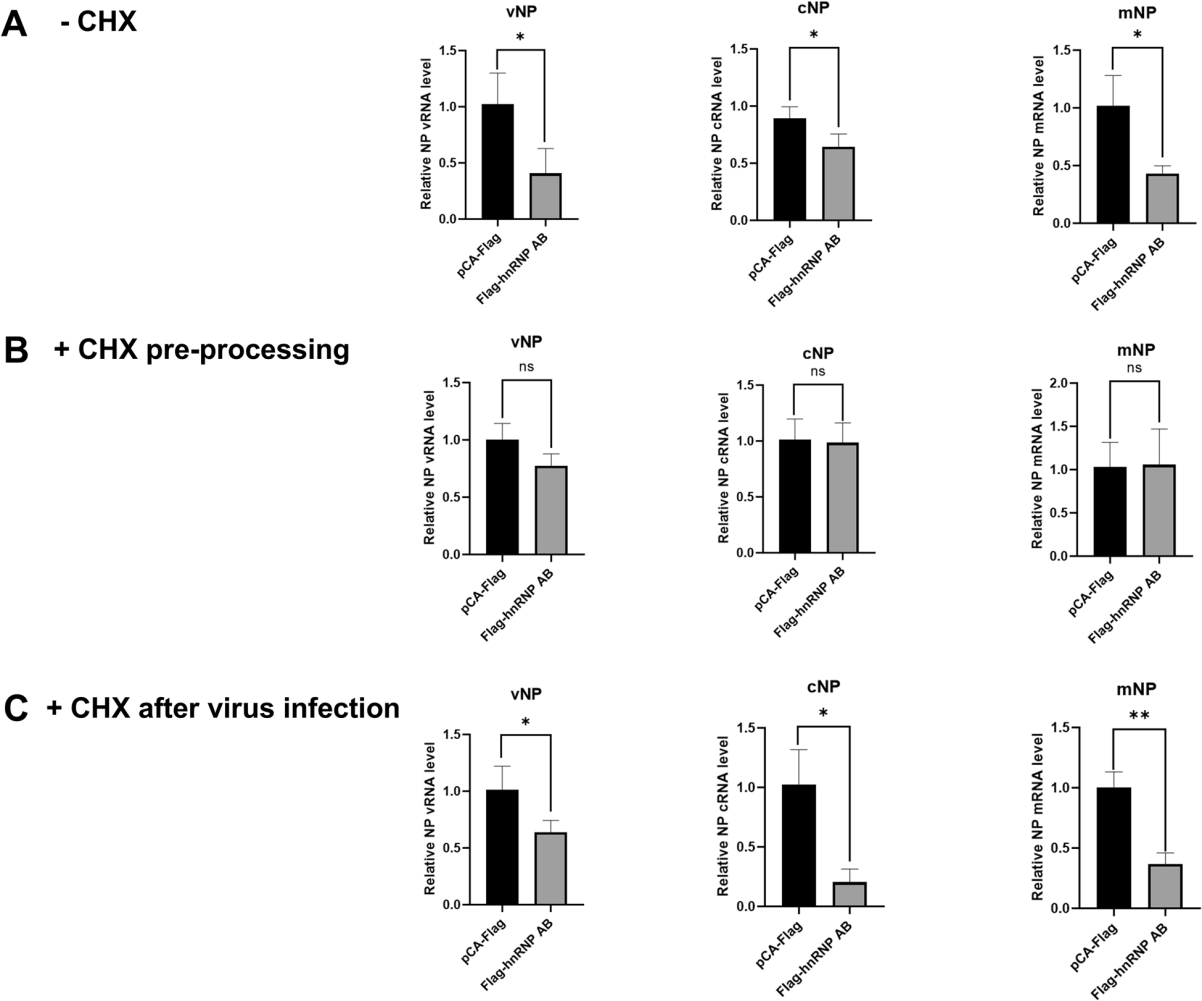
**HnRNP AB inhibits AIV RNA synthesis levels**. **A** HnRNP AB inhibits AIV vRNA synthesis. DF-1 cells were transfected with Flag-hnRNP AB or pCA-Flag for 24 h. Then the transfected cells were infected with A2013-H9N2 (MOI of 0.01) for 8 h. The levels of NP RNAs were detected by qRT-PCR using specific primers for vRNA, cRNA, and mRNA shown in Table 1. **B** HnRNP AB does not inhibits AIV mRNA synthesis in primary transcription. DF-1 cells were transfected with Flag-hnRNP AB or pCA-Flag for 24 h. CHX (100 μg/mL) pre-treated cells for 1 h. Then the transfected cells were infected with A2013-H9N2 (MOI of 0.01) for 8 h. The levels of NP RNAs were detected by qRT-PCR using specific primers for vRNA, cRNA, and mRNA shown in Table 1. **C** HnRNP AB inhibits AIV cRNA synthesis in replication. DF-1 cells were transfected with Flag-hnRNP AB or pCA-Flag for 24 h. Then, cells were infected with A2013-H9N2 (MOI of 0.01), and after 2 h, CHX (100 μg/mL) added to cells. Finally, samples were collected 8 h after viral infection. The levels of NP RNAs were detected by qRT-PCR using specific primers for vRNA, cRNA, and mRNA shown in Table 1. The relative RNA levels were calculated by the 2^−ΔΔCT^ method, including normalization to CT values of GAPDH. The experiment was repeated three times, all by two-tailed Student’s *t* test. * *p* < 0.05, ** *p* < 0.01.

Since hnRNP AB may interfere with the assembly of RdRp by inhibiting the interaction of PB2-PB1, which further affect the synthesis of avian influenza viral RNA [23, 25]. This study investigated the effect of hnRNP AB on the levels of vRNA, cRNA and mRNA of AIV. As shown in Figure 5A, overexpression of hnRNP AB reduced the vRNA, mRNA, and cRNA levels of H9N2 virus.

CHX can block the synthesis of viral and host proteins, and the cRNA will be degraded if it cannot bind to the newly synthesized polymerase subunit and NP [26]. It results in an inability to complete vRNA replication, but has no effect on primary transcription [27, 28]. The effect of hnRNP AB on mRNA levels in AIV primary transcription was further investigated. As shown in Figure 5B, there was no significant difference in RNA levels between the hnRNP AB and the control group. HnRNP AB did not affect the mRNA levels in primary transcription.

Accordingly, the effect of hnRNP AB on cRNA synthesis during AIV replication was explored. RNA levels in the hnRNP AB group were significantly reduced, compared to the control group. It suggested that hnRNP AB inhibited influenza virus replication by reducing cRNA levels (Figure 5C). Notably, mRNA levels were also significantly reduced, which may be due to the replication of the virus affecting primary transcription. In summary, hnRNP AB reduced RNA synthesis during AIV replication, but had no effect on primary transcription.

### HnRNP AB inhibits PB2 mRNA nuclear export by decreasing UAP56-binding mRNA

**Figure 6:**
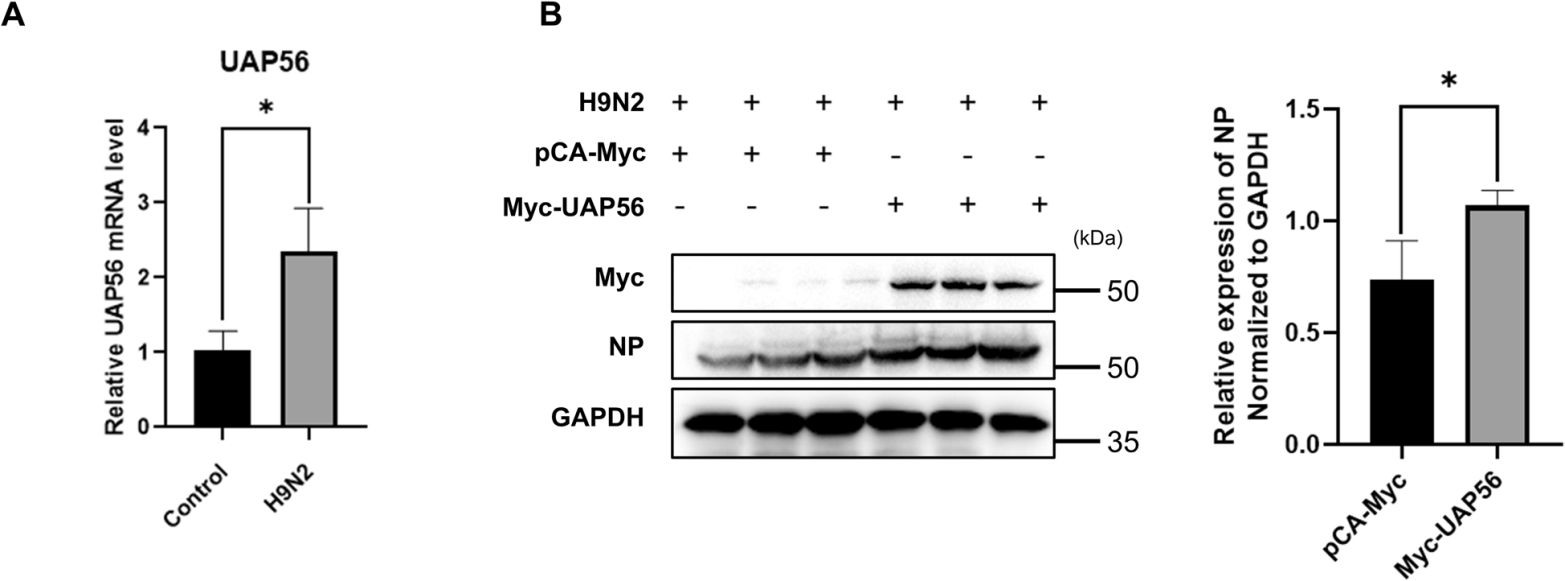
**AIV replication is dependent on the nuclear export factor of the avian host protein UAP56**. **A** The RNA level of UAP56 nuclear export factor increased after DF-1 cell infection. DF-1 cells were infected with A2013-H9N2 virus (MOI of 0.01) for 24 h, the RNA level of UAP56 was detected by qRT-PCR. The relative RNA levels were calculated by the 2^−ΔΔCT^ method, including normalization to CT values of GAPDH. **B** UAP56 promotes viral replication. DF-1 cells were transfected with Myc-UAP56 or pCA-Myc for 24 h. Then DF-1 cells were infected with A2013-H9N2 virus (MOI of 0.01) for 24 h. Cell lysate was collected at 24 hpi. Western blot was used to detect the levels of Myc-UAP56, NP and GAPDH proteins. The experiment was repeated three times, the expression level of viral NP protein was normalized to GAPDH using ImageJ respectively, all by two-tailed Student’s *t* test. * *p* < 0.05.

Previous research data have shown that hnRNP AB can inhibit PB2 protein expression by reducing PB2 mRNA nuclear export [16]. In order to investigate the specific mechanism, we first explored the avian host mRNA nuclear export pathway on which AIV replication depends. It has been reported that the host nuclear export factors UAP56 are often used by viruses to promote viral mRNA export [29–32]. As shown in Figure 6A, the mRNA level of UAP56 in virus-infected cells was significantly higher than that in the control group. Besides, the NP protein in the overexpressed UAP56 group was significantly higher than that in the control group (Figure 6B). It suggested that UAP56 is a key role in H9N2 infection.

**Figure 7:**
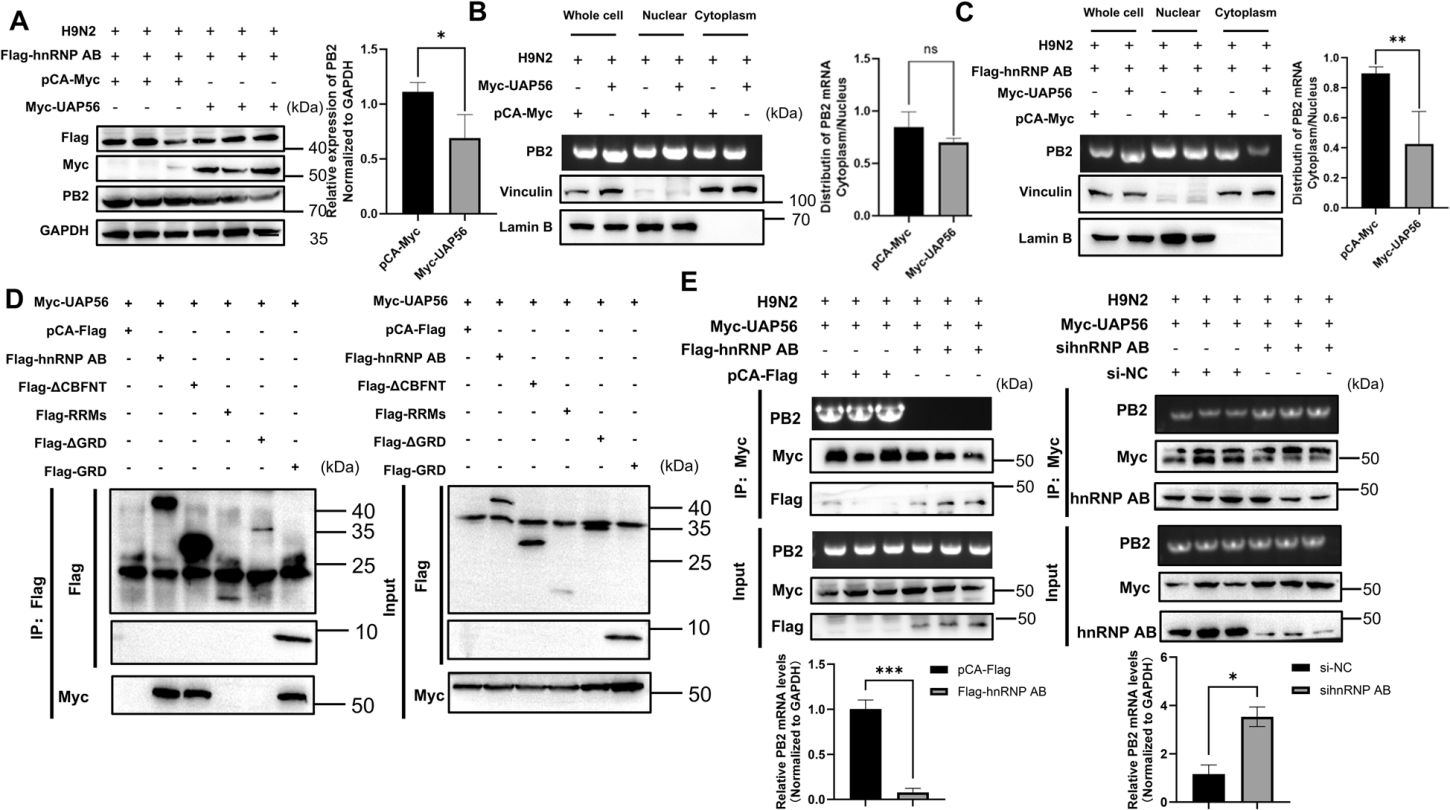
**HnRNP AB inhibits PB2 mRNA nuclear export by decreasing UAP56-binding mRNA**. **A** HnRNP AB reduces PB2 protein via UAP56. DF-1 cells were co-transfected with Flag-hnRNP AB and Myc-UAP56 (pCA-Myc) for 24 h. Then the transfected cells were infected with A2013-H9N2 (MOI of 0.01) for 24 h, cell lysates were collected, and Flag-hnRNP AB, Myc-UAP56, PB2 and GAPDH protein were detected by western blot. **B, C** HnRNP AB inhibits PB2 mRNA nuclear export via UAP56. DF-1 cells were transfected with Myc-UAP56 or pCA-Myc for 24 h. Then the transfected cells were infected with A2013-H9N2 (MOI of 0.01) for 24 h. The cell lysate was collected and separated the nucleus and cytoplasmic fractions. DF-1 cells were co-transfected with Flag-hnRNP AB and Myc-UAP56 (pCA-Myc) for 24 h. Then the transfected cells were infected with A2013-H9N2 (MOI of 0.01) for 24 h. The cell lysate was collected and separated the nucleus and cytoplasmic fractions. Western blot was used to detect the vinculin and Lamin B to check the purity of the separation. RT-PCR was used to detect the mRNA of PB2. **D** HnRNP AB inhibits the binding of UAP56 to PB2 mRNA. 293 T cells were co-transfected with Myc-UAP56 and different truncates of Flag-hnRNP AB (pCA-Flag) for 48 h, respectively. Cell lysates were collected, immunoprecipitated with anti-Flag tag antibody. Western blot was used to detect the Flag-hnRNP AB and Myc-UAP56. **E** DF-1 cells were co-transfected with Myc-UAP56 and Flag-hnRNP AB (pCA-Flag) for 24 h, respectively. And then the transfected cells were infected with A2013-H9N2 (MOI of 0.01) for 24 h, cell lysates were collected, immunoprecipitated with anti-Myc tag antibody. Flag-hnRNP AB and Myc-UAP56 were detected by western blot. RT-PCR and qRT-PCR were used to detect the mRNA of PB2. The relative RNA levels were calculated by the 2^−ΔΔCT^ method normalized to GAPDH. All by two-tailed Student’s *t* test. * *p* < 0.05, ** *p* < 0.01, *** *p* < 0.001.

We further explored whether hnRNP AB inhibited PB2 mRNA nuclear export via UAP56. HnRNP AB and UAP56 were co-expressed in DF-1 cells, then H9N2 virus infected cells. As shown in Figure 7A, PB2 protein level significantly decreased in UAP56 group. It indicated that hnRNP AB inhibited PB2 protein expression through UAP56. Next, the mechanism of the two host proteins involved in the nuclear export of PB2 mRNA was further explored. There was no significant difference in the amount of PB2 mRNA in the nucleus and cytoplasm between the UAP56 group and control group (Figure 7B). We continued to co-express Flag-hnRNP AB and Myc-UAP56 (pCA-Myc) in DF-1 cell to analyze the effect of hnRNP AB and UAP56 on the nucleoplasm distribution of PB2 mRNA. As shown in Figure 7C, the content of PB2 mRNA in the cytoplasm was significantly lower than that of the control group after co-expression of hnRNP AB and UAP56, leading to a significant reduction in the cytosolic-to-nuclear ratio of PB2. That was, hnRNP AB inhibited the nuclear export of PB2 mRNA via UAP56.

HnRNP AB utilizes UAP56 to inhibit PB2 mRNA nuclear export, suggesting that hnRNP AB may interact with UAP56. The interaction between hnRNP AB and UAP56 was further explored here. As shown in Figure 7D, full-length hnRNP AB, ΔCBFNT and GRD domain successfully immunoprecipitated Myc-UAP56, while RRMs or ΔGRD did not interacted with Myc-UAP56. This indicated that GRD domain was the key domain of hnRNP AB interacting with UAP56. UAP56 was involved in mRNA nuclear export by binding to mRNA and then transferred the mRNA to ALY. We wondered if hnRNP AB affected UAP56-binding mRNA. As shown in Figure 7E, the amount of PB2 mRNA binding to UAP56 was significantly reduced in the overexpressing hnRNP AB group, while the amount of PB2 mRNA binding to UAP56 significantly increased using sihnRNP AB, which were consistent with results performed by RIP-qPCR. These results suggested that hnRNP AB attenuated the binding ability of UAP56 to PB2 mRNA.

### HnRNP AB inhibits AIV RNA synthesis via UAP56

**Figure 8:**
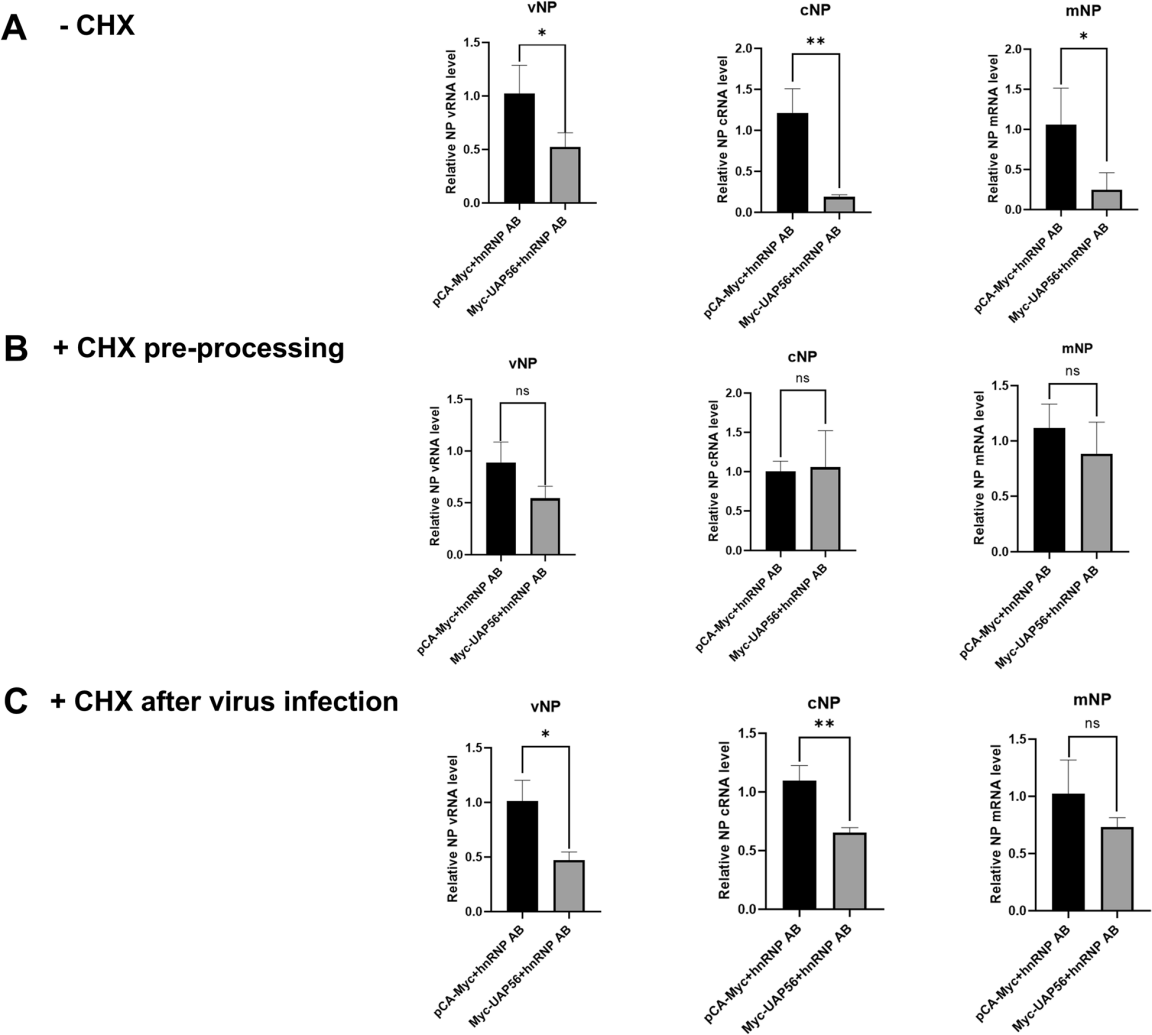
**HnRNP AB inhibits AIV RNA synthesis via UAP56**. **A** HnRNP AB inhibits AIV vRNA synthesis via UAP56. DF-1 cell was co-transfected with Flag-hnRNP AB and Myc-UAP56 or pCA-Myc for 24 h. Then the transfected cells were infected with A2013-H9N2 (MOI of 0.01) for 8 h. The levels of NP RNAs were detected by qRT-PCR using specific primers for vRNA, cRNA, and mRNA shown in Table 1. **B** HnRNP AB does not inhibits AIV mRNA synthesis in primary transcription via UAP56. DF-1 cells were co-transfected with Flag-hnRNP AB and Myc-UAP56 or pCA-Myc for 24 h. CHX (100 μg/mL) pre-treated cells for 1 h. Then the transfected cells were infected with A2013-H9N2 (MOI of 0.01) for 8 h. The levels of NP RNAs were detected by qRT-PCR using specific primers for vRNA, cRNA, and mRNA shown in Table 1. **C** HnRNP AB inhibits AIV cRNA synthesis in replication via UAP56. DF-1 cells were co-transfected with Flag-hnRNP AB and Myc-UAP56 or pCA-Myc for 24 h. Then, cells were infected with with A2013-H9N2 (MOI of 0.01), and after 2 h, CHX (100 μg/mL) added to cells. Finally, samples were collected 8 h after viral infection. The levels of NP RNAs were detected by qRT-PCR using specific primers for vRNA, cRNA, and mRNA shown in Table 1. The relative RNA levels were calculated by the 2^−ΔΔCT^ method, including normalization to CT values of GAPDH. The experiment was repeated three times, all by two-tailed Student’s *t* test. * *p* < 0.05, ** *p* < 0.01.

As shown previously, hnRNP AB inhibited AIV vRNA synthesis (Figure 5C). We further investigated whether hnRNP AB reduced AIV vRNA synthesis by blocking the bind of UAP56 to PB2 mRNA. Co-expression of hnRNP AB and UAP56 decreased H9N2 vRNA, mRNA and cRNA levels (Figure 8A). It suggested that hnRNP AB reduced AIV RNA synthesis via UAP56. The study further investigated the effects of the interaction of hnRNP AB and UAP56 on mRNA levels during primary transcription and cRNA levels during replication of AIV, respectively. Next, cells were pretreated with CHX, and the viral replication process was blocked, but primary transcription was not affected. In the presence of CHX, the results showed that there was no significant difference in mRNA levels between the UAP56 group and the control group, which indicated that hnRNP AB did not reduce the mRNA levels during primary transcription via UAP56 (Figure 8B). The experimental results changed when the cells were infected with AIV and then treated with CHX. The results showed that vRNA and cRNA levels were significantly reduced in the UAP56 group (Figure 8C), indicating that hnRNP AB reduced cRNA levels via UAP56. Thus, hnRNP AB reduced AIV RNA synthesis via UAP56 during replication.

## Discussion

IAV uses cellular mechanisms to complete their life cycle through protein–protein interactions, and interactions also trigger host immune defenses to limit viral infection. HnRNP AB has previously been shown to interact with H9N2 PB2 protein and inhibited viral replication [16]. Subtype H9 AIV has been widely circulating within poultry populations, cases of human infection have already emerged [33–35]. Notably, H9N2 variants have been identified as genetic contributors to the emergence of novel H7N9 and H10N8 strains associated with fatal human infections [36, 37]. We demonstrated that hnRNP AB inhibited replication of poultry-derived H9N2 subtype AIV. As natural reservoirs of AIVs, wild birds serve as crucibles for novel viruses capable of cross-species transmission to domestic poultry, mammals, and humans [38, 39]. The increased migratory activities of wild birds have facilitated more frequent viral reassortment and mutations, with certain variants exhibiting enhanced mammalian adaptation and elevated public health risks [40–42]. It is necessary to investigate the inhibitory effects of hnRNP AB on wild bird-origin AIVs. Our results showed that avian hnRNP AB also had inhibitory effects on wild bird H9, H4 and H13 subtypes of AIV, indicating that hnRNP AB generally inhibited AIV replication.

HnRNP AB belongs to hnRNPs, which are involved in viral life activities [25, 43–47]. For example, hnRNP AB can inhibit IAV replication by interacting with the NP protein of IAV and inhibiting viral mRNA nuclear export [14]. HnRNP AB inhibits IAV polymerase activity, targeting NPs to disrupt the formation of the FulPol complex to inhibit viral replication [15]. Avian hnRNP AB consists of three components. Among them, the N-terminal CBFNT can bind to cis-acting elements and is related to translational regulation. The two RRMs in the middle can recognize and bind RNA. The GRD located at the C-terminus can interact with other proteins. GRD is characterised by an Arg-Gly-Gly tripeptide repeat cluster. Many Arg-Gly structural domains may confer non-specific RNA binding activity to protein or mediate protein–protein interactions [48]. This study indicated that GRD was the functional domain that inhibited AIV. Similarly, the GRD of the human hnRNP AB is the functional domain inhibiting the replication of IAV [14]. The GRD of hnRNP AB can binds to poly(A)^+^ RNA. Based on the data we suggested that hnRNP AB may inhibit viral replication by interacting with mRNAs or proteins via GRD. We demonstrated the interaction between the C-terminus of PB2 and GRD. The C-terminal domain of PB2 includes the 627-domain and the nuclear localization domain (NLD) [49–53]. The C-terminus of PB2 also interacts with PB1 [24, 51]. Host proteins target PB2 to restrict RdRp formation and inhibit viral replication. For example, BAG6 competitively inhibits the assembly of the RdRp complex and induces ubiquitinated degradation of PB2 by interacting with PB2 to limit IAV replication [54]. We demonstrated that hnRNP AB inhibited PB2-PB1 interaction, which may affect RdRp stability. In the nucleus, viral RNA transcription and replication occur in a viral RdRp-dependent manner. The results showed that hnRNP AB reduced viral RNA synthesis and consequently inhibited viral replication. However, our results localize the site of action to the 627-domain and NLD, the specific interaction site of hnRNP AB with PB2 needs to be further explored. The 627-domain of PB2 is associated with viral pathogenicity and host range and affects cRNA formation in infected cells [55–58]. NLD can instruct PB2 protein to enter the nucleus. It implies that hnRNP AB may also inhibit AIV replication through other mechanisms by interacting with the C-terminus of PB2.

Previously we found that hnRNP AB inhibited PB2 protein expression by affecting the nucleoplasmic distribution of PB2 mRNA [16]. Based on this discovery, we found that hnRNP AB blocked the ability of UAP56 to bind mRNA to inhibit the nuclear export of PB2 mRNA. HnRNPs have been reported to mediate viral mRNA nuclear export. For example, hnRNP A1 is used to reduce splicing rates sufficiently for the unspliced and partially spliced HIV viral mRNAs to accumulate in the nucleus [59]. HnRNP A2/B1 may inhibit IAV replication by suppressing NS1 RNA/protein levels and nucleocytoplasmic translocation of NS1 mRNA [14]. Here, we demonstrate that hnRNP AB can inhibit viral replication by suppressing the physiological function of the host mRNA export factor UAP56. UAP56 belongs to TREX, which interacts with pre-mRNA as a splicing factor and subsequently recruits ALY to mRNA, playing an important role in mRNA nuclear export [60–63]. UAP56 contributed to AIV replication. However, hnRNP AB interacted with UAP56 and suppressed its ability to bind viral mRNA, thereby negatively regulating AIV replication. Our study provides a new reference for the mechanism whereby hnRNPs co-opt host cellular proteins to subvert the nuclear export system of viral mRNA.

In conclusion, we demonstrate that the avian hnRNP AB inhibits RdRp assembly by interacting with PB2, and hnRNP AB blocks the ability of UAP56 to bind PB2 mRNA to inhibit PB2 mRNA nuclear export, which in turn affects the normal formation of RdRp and reduces AIV RNA synthesis, thereby inhibiting viral replication.

## Data Availability

All data generated during this study is provided within the manuscript.
